# Versatile and sensitive dual-detection HPLC method for rapid quantification of apomorphine hydrochloride: application to ex vivo percutaneous permeation and deposition studies utilising novel transdermal pressure-sensitive adhesive patches

**DOI:** 10.1007/s13346-025-01910-z

**Published:** 2025-07-15

**Authors:** Andrew P. Graham, Qonita Kurnia Anjani, René Holm, Alejandro J. Paredes, Ryan F. Donnelly

**Affiliations:** 1 School of Pharmacy, Medical Biology Centre, Queen’s University Belfast, 97 Lisburn Road, Belfast, BT9 7BL UK; 2 Department of Physics, Chemistry and Pharmacy, University of Southern Denmark, Campusvej 55, Odense, 5230 Denmark; 3 Chair in Pharmaceutical Technology, School of Pharmacy, Medical Biology Centre, Queen’s University Belfast, 97 Lisburn Road, Belfast, BT9 7BL Northern Ireland, UK

**Keywords:** Apomorphine hydrochloride, HPLC, UV, Fluorescence, Ex vivo, Transdermal pressure-sensitive adhesive patches

## Abstract

Conventional delivery of apomorphine hydrochloride (APO) for management of ‘OFF’ periods in Parkinson’s disease is limited to subcutaneous or sublingual delivery, and would substantially benefit from a transdermal drug delivery system (TDDS). Minimal progression has been made thus far in this area, with current literature presenting debatably flawed or inconsistent experimental design for ex vivo percutaneous permeation and deposition studies, a crucial step in understanding TDDS performance. Provided here is an improved, robust, versatile and easily-transferred analytical methodology, fully validated in accordance with guidelines provided by the International Council for Harmonization of Technical Requirements for Pharmaceuticals for Human Use (ICH). Reversed-phase high performance liquid chromatography (RP-HPLC) is employed with ultraviolet-visible (UV) light or fluorescence intensity (FID) detection in conjunction with a Franz diffusion-cell setup that ensures stability of APO and uses physiologically relevant neonatal porcine skin. Thus, this methodology may be reliably employed by future researchers developing an APO TDDS. To demonstrate application, a superficial investigation into model pressure-sensitive adhesive (PSA) patch systems provided sustained percutaneous delivery of up to 4.015 ± 2.361 µg/cm^2^/hr APO over a 24-hour period. These offer brief insight into a system that has yet to be studied in-depth for percutaneous delivery of APO, and highlight the importance of alternative penetration enhancers or enhancement techniques in developing a TDDS with clinical potential.

**Graphical abstract:**

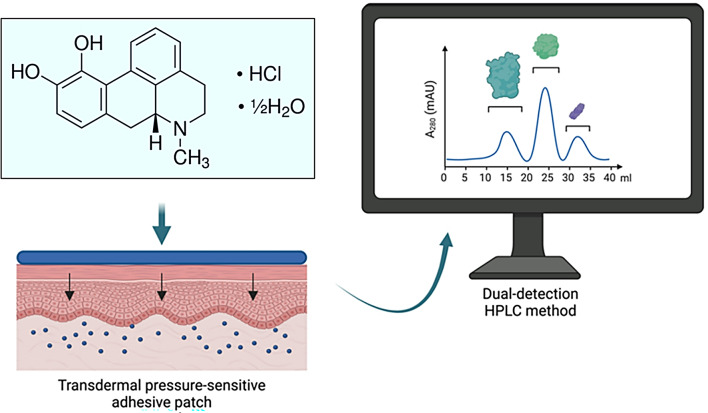

## Introduction

First synthesised by Matthiessen and Wright in 1868, apomorphine hydrochloride (APO) is a highly versatile and potent molecule with a rich history spanning several centuries of pharmacological applications including emesis, sedation and penile induction [1, 2]. Though it is primarily used as a highly efficacious antiparkinsonian agent, mainly for management of ‘OFF’ periods, owing to its broad-spectrum agonistic affinity for dopamine receptors, in addition to several serotonergic and α-adrenergic receptor subtypes. Interestingly, this pharmacological profile is entirely unique to APO [3]. It is in fact the only current alternative to levodopa with equivalent antiparkinsonian efficacy, making it a crucial therapeutic in the armamentarium available to patients suffering from Parkinson’s disease (PD) [1].

Unfortunately, a highly pronounced first-pass effect due to liver metabolism has severely limited conventional delivery of APO to subcutaneous injection intermittently (APO-go^®^; Britannia Pharmaceuticals Ltd.) or continuous infusion *via* a pneumatic pump, with recent FDA approval of a novel sublingual film (Kynmobi^®^; Sunovion Pharmaceuticals Inc.) in 2020 [4, 5]. These methods of administration possess a number of concomitant issues. APO has a short half-life of around 33 min, meaning intermittent use may require a high dosage frequency [6]. Subcutaneous injection of APO may be painful or cause injection-site reactions, such as subcutaneous nodules, and may induce fear in 3.5–20% of patients [7–9]. Moreover, poor patient dexterity during OFF periods causes subcutaneous injections or infusions to be difficult to self-operate [1, 10]. On the other hand, sublingual films are more convenient as they require less precision and skill, however, a high discontinuation rate was shown during phase 3 clinical trials due to frequent oropharyngeal side effects, and Kynmobi^®^ has even been discontinued in the US and Canada as of June 2023 [11–13]. Clearly, further research must focus on alternative delivery methods to help avoid these aforementioned issues and provide superior treatment options that are more acceptable to patients with PD.

Transdermal drug delivery systems (TDDSs) may be an effective alternative, as they bypass first-pass metabolism, are painless, needle-free, easy to self-administer and avoid any oropharyngeal side effects. They have a wider availability of application sites and can provide sustained release of a drug, reducing the dosage frequency per square area of the human body surface, thus decreasing administration site reactions [14]. With this in mind, it is surprising there have only been a handful of publications in the previous 30 years describing various approaches to APO TDDSs including emulsions, iontophoresis, ion-exchange, ion-pairing and microneedles [15–24]. In fact, only a few of these studies have explored transdermal delivery of APO in humans, with relatively promising findings [17, 22, 23]. Indeed, research in this area has seen minimal progression and is stalling in the preclinical stages. This is despite groundbreaking advances in TDDSs in recent decades, with novel technologies such as microneedles being named one of the “Top 10 Emerging Technology of 2020” by the World Economic Forum [14, 25]. With new approaches, and the potential benefits for patients with PD, the future prospectives for development of an APO TDDS are promising.

Ex vivo percutaneous permeation and deposition studies are essential to the development of a successful APO TDDS. Indeed, these are included in many of the aforementioned publications yet, upon review, a distinct lack of consistent and robust methodology is evident. For instance, many studies previously employed skin tissues with poor physiological relevancy sourced from rodents or snakes [15, 16, 20, 24, 26]; or use of challenging-to-obtain and costly human skin [18, 21, 24, 26]. Ideally, an ex vivo experimental protocol should make consistent use of cheap, easily obtained, physiologically relevant tissues such as, neonatal porcine skin. Additionally, due to the rapid auto-oxidation of APO in aqueous environments, verification of sufficient stability of APO within the release media, diluent or storage solution is crucial, however, several studies relied on sub-optimal antioxidant strategies leading to questionable stability of APO during ex vivo studies, based on a recent study by Ang et al. in 2016 [15, 18, 21, 24, 27]. Furthermore, as these samples would contain proteins, lipids, and other matrix substances from skin tissue, the quantitative analytical procedure should be validated in accordance with the “International Council for Harmonization of Technical Requirements for Pharmaceuticals for Human Use” (ICH) M10 bioanalytical guidelines, yet this is not the case for any of the currently published methods in literature [15, 16, 18, 19, 21, 24, 28].

Therefore, the aim of this work was to provide an improved methodology for researchers in this field, with a rapid reversed-phase high performance liquid chromatography (RP-HPLC) method fully validated in-line with ICH M10 guidelines that is robust, flexible and easily-transferred due to dual-quantification capability by ultraviolet-visible (UV) absorbance or fluorescence intensity detection (FID). Herein, a novel drug-in-adhesive pressure-sensitive adhesive (PSA) patch for APO was applied as a model TDDS to verify utility of an ex vivo percutaneous permeation and deposition experimental protocol that employs Franz diffusion cell apparatus with a biological membrane composed of physiologically relevant, low cost and easy-to-obtain neonatal porcine skin. Through inclusion of ascorbic acid as an effective antioxidant, stability of APO may be more reliably maintained throughout the study [26].

## Materials and methods

### Materials

R - (-) - Apomorphine hydrochloride hemihydrate (APO), phosphate buffered saline (PBS) pH 7.4 tablets, trifluoroacetic acid (TFA), poly(ethylene glycol) (PEG), acetonitrile (ACN), ethyl acetate (EA) and methanol (MeOH) (each of HPLC grade or higher) were supplied by Sigma-Aldrich, Darmstadt, Germany. Ascorbic acid (AA) was purchased from Alfa Aesar, Massachusetts, USA. Veet^®^ hair removal cream was manufactured by Reckitt Benckiser Group PLC, Slough, UK and polytetrafluoroethylene (PTFE) 0.45 μm filters were obtained from Agilent Technologies, California, USA. DuroTak^®^ 87-2677, a polyacrylic PSA functionalised with carboxyl groups, was supplied by Henkel, Düsseldorf, Germany. Skin was obtained from stillborn neonatal piglets that were frozen at -20 °C within a 24-hour period after farrowing, then thawed prior to harvest of skin by scalpel or Padgett^®^ Model B electric dermatome (Integra Life Sciences Corp., Ratingen, Germany).

### Chromatographic conditions

The HPLC instrument model was an Agilent 1260 Infinity II series equipped with a 1260 Quat Pump VWD, FLD and Vialsampler. An Osaka Soda Capcell PAK C18 (150 × 4.6 mm, 5 μm) (Osaka Soda Co. Ltd., Okayama, Japan) fitted with Phenomenex SecurityGuard™ analytical guard cartridge (Phenomenex Ltd., Macclesfield, UK), was employed and operated without temperature control. Aqueous (A) and organic (B) mobile phases were 0.1% (v/v) TFA in water and ACN respectively, and used in an isocratic 70:30 (A: B) ratio prepared *via* online mixing. The flow-rate was set to 1.0 mL/min and the injection volume was 20 µL. During analysis, UV-Vis and excitation wavelength was 274 nm. Emission wavelength was 458 nm, with PMT gain set to 10 V. Run-time per injection was 5 min. Samples were dissolved in 0.1% (w/w) AA in PBS pH 7.4.

### Preparation of neonatal porcine skin extract, stock solution and working standards

A 250 mg segment of full thickness neonatal porcine skin was homogenised at 50 osc/s for 10 min using a TissueLyser LT, in 1.5 mL of 0.1% (w/w) AA in PBS pH 7.4. Following this, the homogenate was then transferred to 50 mL of the same solvent and incubated at 37 °C for 24 h. The extract was then filtered *via* a 0.45 μm PTFE filter and stored in a refrigerator prior to use.

On the day of analysis, a fresh 1 mg/mL bulk solution of APO was prepared by dissolving 25 mg in 2 mL of MeOH, then making up to volume with 0.1% (w/w) AA in PBS pH 7.4. Subsequently, a 0.1 mL aliquot was diluted in 0.9 mL of the neonatal porcine skin extract to prepare a 0.1 mg/mL stock. Working standards were then prepared *via* serial dilutions.

### Determination of UV and FID detection parameters

A 10 µg/mL APO solution was prepared in a diluent composed of a 70:30 ratio of 0.1% (v/v) TFA in water, and ACN respectively. Then, 0.3 mL aliquots were transferred in triplicate to a flat-bottom 96-well plate (Greiner Bio-One, Kremsmünster, Austria), and scanned between 220 and 800 nm at room temperature using a Fluostar Omega spectrometer (BMG LABTECH, Ortenberg, Germany). The obtained spectrum was background corrected with blank diluent.

A fixed-excitation emission wavelength scan was performed in triplicate between 300 and 800 nm on a 0.1 µg/mL APO solution and blank diluent, using an F-2710 FL spectrophotometer (Hitachi High Technologies America Inc., Illinois, USA) with λ_exc_ set to 274 nm. Photomultiplier tube (PMT) gain was set to 400 V.

### Method validation

Analytical method validation parameters, guidelines and acceptance criteria were derived from ICH M10 bioanalytical guidelines [28].

#### Linearity and range

Linearity was determined by analysing in triplicate over three days, three calibration curves containing at least six non-zero calibrators in the following ranges of APO: 12.5–0.098 µg/mL and 12.5–0.391 µg/mL for FID and UV, respectively. Simple linear regression models were constructed *via* 1/x weighting, based on the average peak area response of the calibration standards, then employed to determine the acceptability of each curve based on the accuracy and precision (% bias and % coefficient of variation (CV), respectively; see Eqs. (1) and (2)) of the back-calculated concentrations of each standard. The acceptance limits derived from ICH M10 were ± 15% bias or CV at each concentration, all except the lowest non-zero calibration standard (LLOQ) which was ± 20%.

1 $$\% {\rm{ Bias }} = \left( {{{{C_m} - {C_t}} \over {{C_t}}}} \right) \times 100$$

where; C_m_ is the measured or obtained concentration of APO and C_t_ is the true or actual concentration of APO.

2 $$\% CV = \left( {{{{\sigma _{{C_m}}}} \over {{\rm{ Mean }} \ {C_m}}}} \right) \times 100$$

where; $\:{\sigma\:}_{{C}_{m}}$ is the standard deviation of the measured or obtained concentration of APO

#### Specificity

Specificity from degradants and other interfering species derived from neonatal porcine skin extract, was confirmed through visual analysis of corresponding chromatograms in the retention time region of APO. Degradants were generated through storage of a 100 µg/mL APO solution in PBS (pH 7.4) at 37 °C for 7 days, without the presence of any stabiliser.

#### Selectivity

Blank neonatal porcine skin extract and LLOQ calibration standard were analysed in sextuplicate, with peak area responses used to determine the LLOQ: matrix ratio; see Eq. (3). Acceptance criteria required an LLOQ: matrix ratio of > 5.

3 $${\rm{LLOQ: Matrix }} = {{P{A_{LLOQ}}} \over {P{A_{{\rm{matrix }}}}}}$$

where; $\:{PA}_{LLOQ}$ is the peak area of the respective LLOQ QC standard and $\:{PA}_{matrix}$ is the peak area of the blank neonatal porcine extract in the retention time region of APO.

#### Within-run and between-run accuracy and precision

Accuracy and precision of the analytical method was assessed using % bias and % CV, respectively, across each calibration range at four concentration levels. These were each analysed in quintuplicate in three separate runs across three consecutive days. Assessment of within-run performance was determined through analysis of data obtained on day 1, as opposed to between-run performance which took into account the entire dataset from days 1–3. Acceptance limits were ± 15% bias or CV (± 20% for LLOQ).

#### Carryover

Carryover of analyte between injections was determined by injecting 50 µg/mL APO, followed by a subsequent injection of blank neonatal porcine skin extract. This process was performed in triplicate, with acceptance criteria requiring any response in the retention time region of APO to be < 20% of the average LLOQ response achieved during Sect. Within-run and between-run accuracy and precision. Thus, LLOQ: matrix ratio was required to remain > 5.

#### Dilution integrity

Dilution integrity was assessed using arbitrary dilution factors of 10 and 100, from the 0.1 mg/mL APO stock. The accuracy and precision of the diluted solutions were required to be within ± 15% bias and CV respectively. The measurements were performed in triplicate.

#### Robustness

Various method parameters, namely: flow-rate (mL/min); mobile phase composition (% v/v) and; % (v/v) TFA concentration in the aqueous phase; were purposefully altered to explore how this may affect performance of the analytical method. Method performance was assessed based on % recovery (calculated using Eq. (4)) and retention time of a 10 µg/mL APO standard, with measurements performed in quintuplicate.

4 $$\% \ {\rm{ Recovery }} = \left( {{{{C_m}} \over {{C_t}}}} \right) \times 100$$

### Stability studies

Stability of APO in PBS (pH 7.4) with and without 0.1% (w/w) AA was determined over a period of 28 days in various storage conditions, including: 37 °C, room temperature (lit and dark conditions), fridge (2–8 °C), and freezer ( -20 °C). A high and low concentration of APO, 100 µg/mL and 10 µg/mL respectively, were both sampled and analysed in triplicate on days 0, 1, 2, 3, 7, 14, 21 and 28. The relative stability of APO at each timepoint was determined based upon the average % recovery (see Eq. 4) from the initial concentration on day 0.

### Saturation solubility

In quadruplicate, a known excess of APO was added to 1 mL of PBS (pH 7.4) with 0.1% (w/w) AA and subsequently vortexed for 60 s prior to incubation at 37 °C for 24 h in an ISF-7100 orbital incubator (Jeio Tech, Daejeon, South Korea) operating at 40 RPM. The solution was then filtered with a 0.45 μm PTFE filter prior to centrifugation at 14,000 RPM for 10 min. The supernatant was sampled, diluted and analysed using the validated RP-HPLC method.

### Extraction of APO from porcine skin

**Fig. 1:**
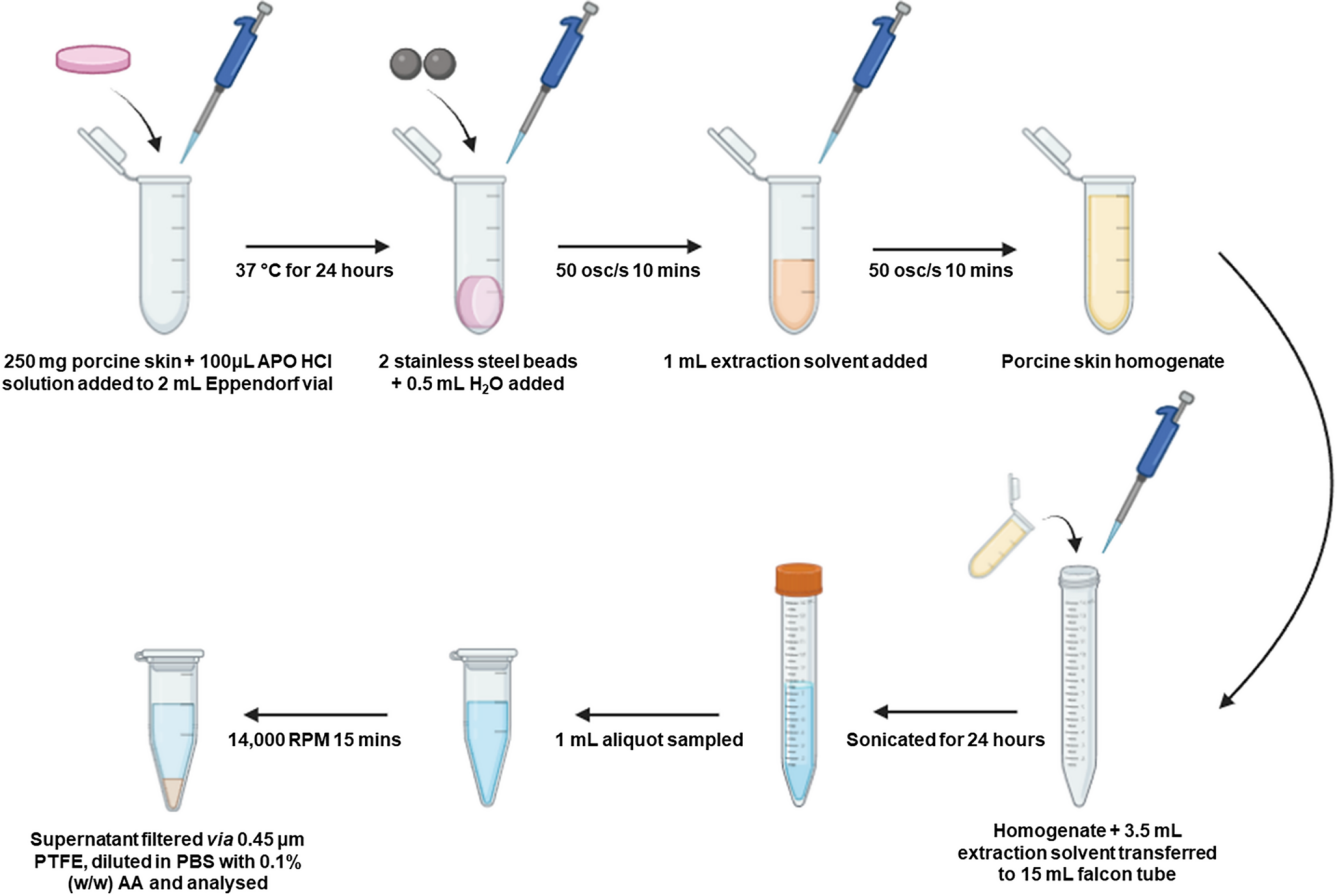
Schematic illustration of procedure for extraction of APO from neonatal porcine skin

For quantification of APO within the skin, several solvents were trialled based on extraction efficiency *via* a solid-liquid extraction method, outlined in Fig. 1. Namely: 0.1% (w/w) AA in water, MeOH and ACN. These solvents were assessed in quintuplicate on skin samples containing either 100 µg–10 µg of APO, by incubating a 2 mL Eppendorf vial of 250 ± 10 mg of full thickness skin in 100 µL of either 1 mg/mL or 0.1 mg/mL solution for 24 h at 37 °C. Subsequently, 0.5 mL of 0.1% (w/w) AA in water and two 5 mm diameter stainless steel beads were added to the same vial, and the contents homogenised at 50 Hz for 10 min using a TissueLyser LT (Qiagen N.V., Hilden, Germany). Prior to another cycle of 10 min at 50 Hz, 1 mL of extraction solvent was added. The homogenate was transferred to a 15 mL falcon tube, to which an additional 3.5 mL of the corresponding extraction solvent was added. This was then sonicated using a QS25 Ultrasonic bath (Ultrawave Ltd., Cardiff, UK) for 24 h. A 1 mL aliquot was centrifuged at 14,000 RPM for 15 min, and the supernatant filtered using a 0.45 μm PTFE filter. Filtrate was diluted in PBS (pH 7.4) with 0.1% (w/w) AA prior to analysis by the validated RP-HPLC method.

### Preparation of APO drug-in-adhesive PSA films

Blends were initially prepared by dissolving DuroTak ^®^ 87-2677 in ethyl acetate by dual-assymetric centrifugation mixing at 3000 RPM for 2.5 min in a FlackTek™ DAC 330 − 100 SE SpeedMixer^®^ (FlackTek Inc., Carolina, USA) then adding the remainder of the respective components along with five 3 mm diameter zirconium oxide beads (Simply Bearings Ltd., Leigh, UK) and mixing at 3500 RPM for a further 5 min. Full compositions of each mixture are outlined in Table 1. The final blends were then sonicated for 2 hours prior to casting 5 g into a 100 × 30 mm steel frame affixed to a Perspex base coated with a silicon sheet. Films were dried for 24 h in a fume hood prior to excision by scalpel into 10 × 10 mm segments. Based on the blend compositions, theoretical loading for each film formulation was 5 mg/cm^2^ and 8.33 mg/cm^2^ for APO and PEG penetration enhancers, respectively.

**Table 1:** Full compositions of solvent blends for Preparation of APO PSA films

Formulation				
	F1	F2	F3	F4
Component	% (w/w)			
DuroTak ^®^ 87-2677	25	25	25	25
EA	71	66	66	66
APO	3	3	3	3
AA	1	1	1	1
PEG 200	-	5	-	-
PEG 400	-	-	5	-
PEG 3000	-	-	-	5

### Determination of drug distribution of APO PSA films via multi-photon microscopy

APO PSA films were visualised using a TCS SP8 MP laser scanning multi-photon microimaging system (Leica Microsystems Ltd., Wetzlar, Germany) fitted with an HC PL APO 10x/0.40 DRY objective, in combination with a Mai Tai^®^ eHP DeepSee™ (MKS Instruments Inc., Massachusetts, USA) infrared laser excitation source. The sample was excited at a wavelength of 822 nm, and detected using a 429–468 nm internal spectral HyD detector, with a scan speed of 600 Hz and pixel dwell-time of 0.2 µs.

### Ex vivo permeation and deposition of APO PSA films

**Fig. 2:**
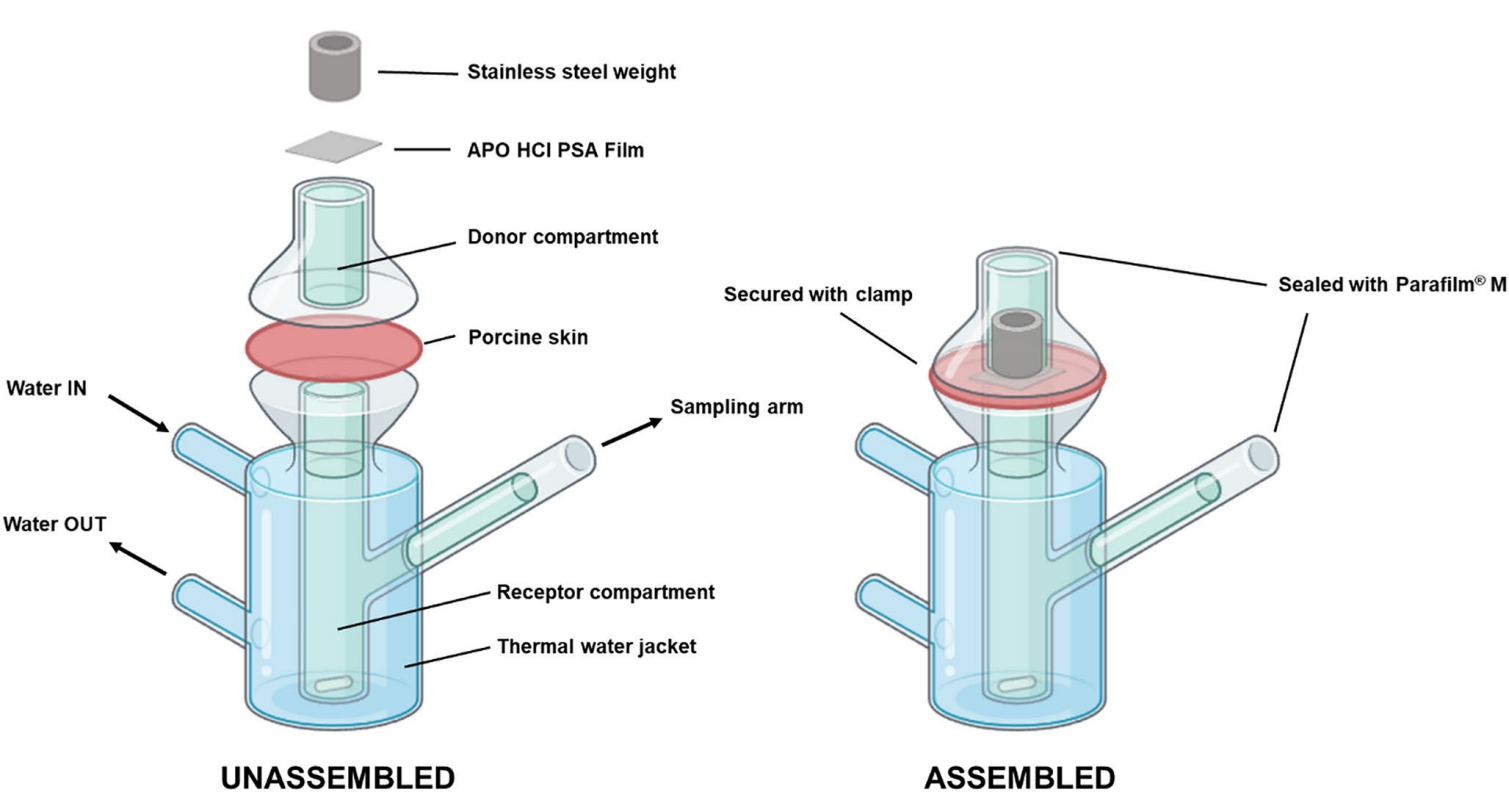
Schematic diagram of Franz diffusion cell apparatus setup employed during ex vivo permeation and deposition studies using dermatomed neonatal porcine skin

The experimental setup for the ex vivo percutaneous permeation and deposition studies is displayed in Fig. 2. Initially, dermatomed skin of ~ 350 μm in thickness was treated with Veet^®^ hair removal cream, rinsed and then equilibrated in PBS (pH 7.4) with 0.1% (w/w) AA for 30 min. A single 10 × 10 mm APO PSA film was applied per diffusion cell using a syringe plug operated manually, with a 5.0 g stainless steel weight then placed atop. The skin was then mounted onto Franz diffusion cell apparatus of 1.77 cm^2^ diffusional area, with the *stratum corneum* facing the donor compartment. The receptor compartment contained 12 mL of PBS (pH 7.4) with 0.1% (w/w) AA as release media, that was temperature-controlled at 37.4 °C using a thermal water jacket and stirred at 600 RPM. At defined time intervals (1, 2, 3, 4, 6 and 24 h), 200 µL aliquots were sampled from the receptor compartment and replaced with fresh media. These were centrifuged at 15,300 RPM for 10 min prior to the supernatant being collected for analysis *via* the validated RP-HPLC method. Upon experiment completion, the skin was collected and processed as outlined in Sect. Extraction of APO from porcine skin in order to determine the amount of APO deposited within the skin. All samples were stored at 2–8 °C until analysis.

### Statistical analysis

Calculation of mean, standard deviation, bias, coefficient of variation, and simple linear regression was performed used Microsoft ^®^ Excel ^®^ version 2409 (Microsoft Corporation, Redmond, USA). All one-way or two-way ANOVA statistical analysis was performed using GraphPad Prism version 10.3.1 (GraphPad Software Inc., California, USA). Results were considered significant if *p* < 0.05. * *p* < 0.05, ** *p* < 0.01, *** *p* < 0.001, **** *p* < 0.0001.

## Results and discussion

### UV and FID detection parameters

**Fig. 3:**
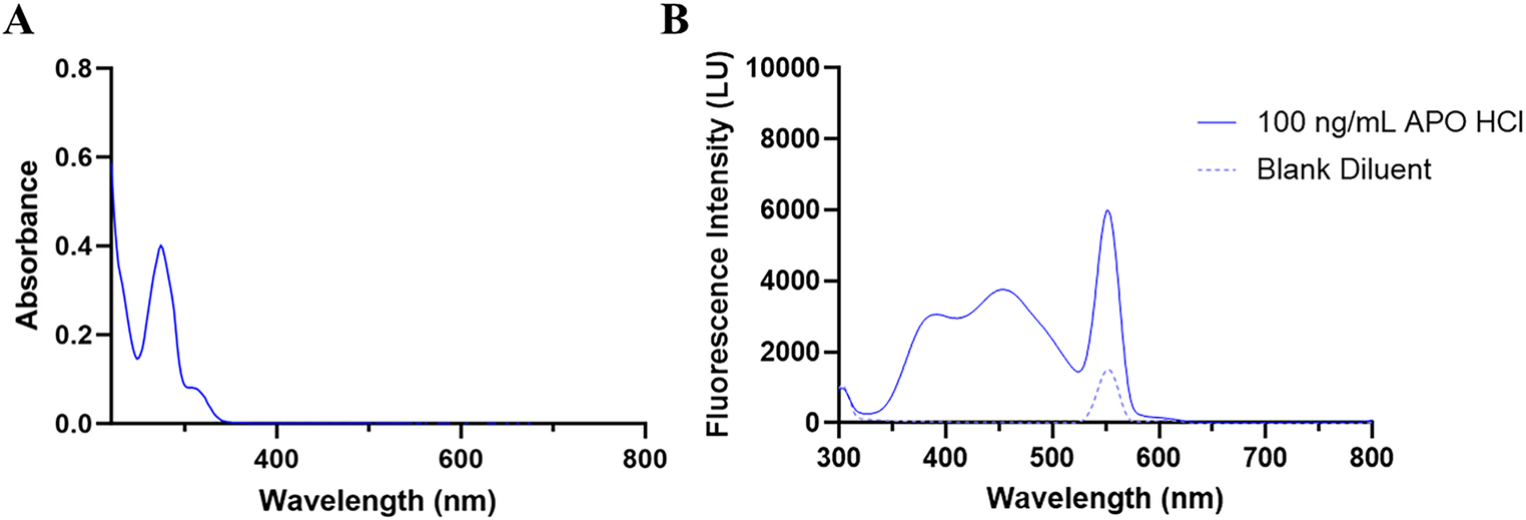
(**A**) Absorption spectra of APO dissolved in 70:30 (v/v) 0.1% (v/v) TFA in water and ACN. Mean measurements shown (*n* = 3). (**B**) Emission spectra of APO dissolved in 70:30 (v/v) 0.1% (v/v) TFA in water and ACN. Generated using a fixed-excitation wavelength of 274 nm with a PMT gain of 400 V. Mean measurements shown (*n* = 3)

UV and FID spectrophotometry were each employed to identify suitable detection parameters that provide optimum sensitivity of the RP-HPLC method. The UV spectrum of APO prepared in the mobile phase is shown in Fig. 3A, confirming that the λ_max_ was 274 nm, in line with previously reported literature [16, 19, 20, 27, 29, 30]. Additionally, APO is known to be fluorescent, providing an excellent opportunity to improve sensitivity and selectivity [24, 31, 32]. A fixed-excitation emission scan with 274 nm set as the excitation wavelength revealed a selective region of emission between approximately 350–525 nm, as shown in Fig. 3B. The wavelength of maximum emission in this region was 458 nm. Similarly, this was in line with previous reports [18, 33]. These detection parameters were thus employed in the developed RP-HPLC method.

### Method validation

#### Linearity and range

**Fig. 4:**
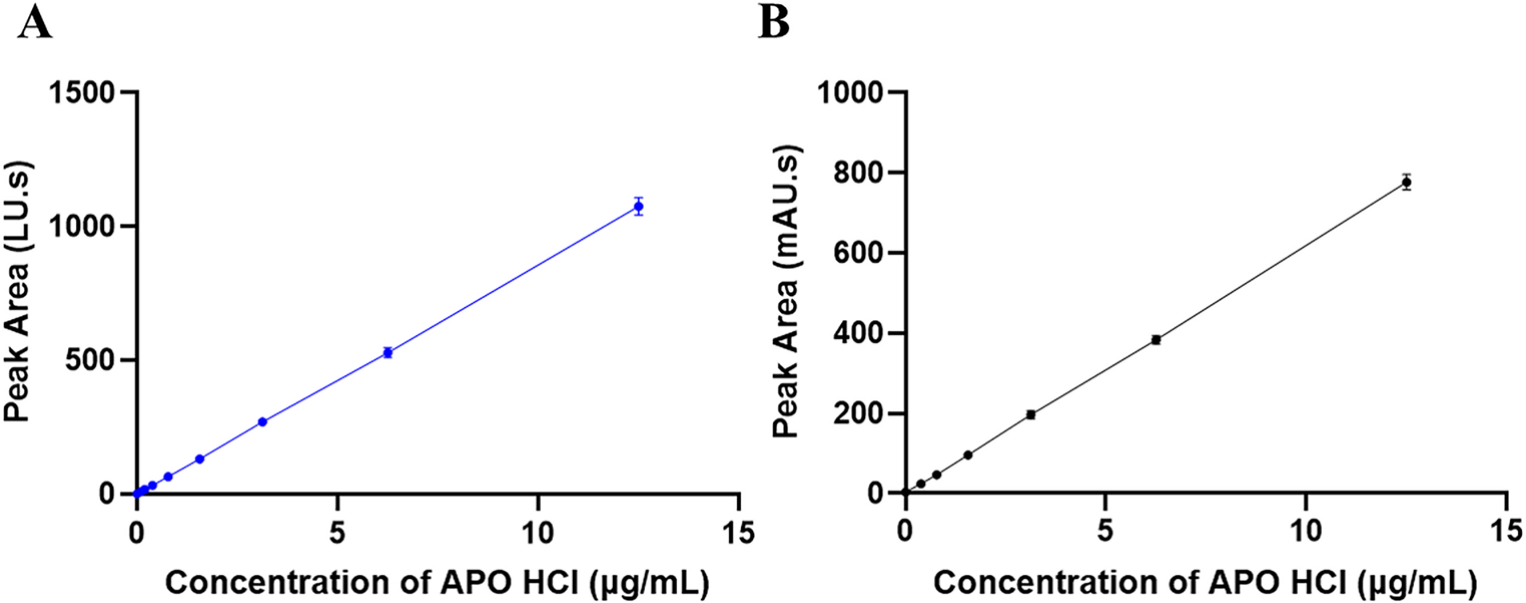
(**A**) FID calibration curve of APO prepared in porcine skin extract in range of 12.5–0.098 µg/mL. (Means ± SD, *n* = 3). (**B**) UV calibration curve of APO prepared in porcine skin extract in range of 12.5–0.391 µg/mL (Means ± SD, *n* = 3)

In line with ICH M10 bioanalytical guidelines, linearity and range of the RP-HPLC method were determined using a minimum of 6 non-zero calibration standards between 12.5 and 0.098 µg/mL and 12.5–0.391 µg/mL, for FID and UV respectively [28]. The linear regression models were constructed based on average peak area and plotted in Fig. 4. Summaries are provided in Table 2, with R^2^ values > 0.9999 confirming sufficient linearity is achieved *via* both detection techniques. Furthermore, these calibration curves were deemed acceptable, as shown in Table 3. For both FID and UV, bias and CV values were within ± 15% for each calibration standard. In exception to this was the LLOQ for FID, which was within ± 20% bias.

**Table 2:** Summary of linear regression analysis of calibration curves shown in Fig. 4

Detection	Slope	Y-intercept	*R* ^2^	ULOQ (ug/mL)	LLOQ (ug/mL)
FID	85.994	-1.699	0.9999	12.5	0.098
UV	61.990	0.614	0.9999	12.5	0.391

**Table 3:** Back-calculated concentrations of APO based on linear regression models summarised in Table 1 (Means ± SD, *n =* 3)

	FID			UV		
Concentration of APO (µg/mL)	Peak Area (LU.s)	Bias (%)	CV (%)	Peak Area (mAU.s)	Bias (%)	CV (%)
12.500	1141.27 ± 8.63	-0.39	0.75	819.26 ± 6.23	-0.13	0.76
6.250	565.84 ± 7.48	-1.00	1.32	405.92 ± 6.96	-0.81	1.71
3.125	282.40 ± 3.87	-0.74	1.36	203.37 ± 3.56	-0.19	1.74
1.563	141.29 ± 3.29	0.20	2.29	102.10 ± 2.43	1.08	2.34
0.781	69.72 ± 2.69	0.68	3.72	50.59 ± 1.78	1.90	3.40
0.391	34.79 ± 1.61	3.99	4.31	23.13 ± 2.60	-3.09	10.45
0.195	17.28 ± 0.92	10.43	4.64	- - -	-	-
0.098	8.15 ± 1.30	19.03	12.19	- - -	-	-

#### Specificity

**Fig. 5:**
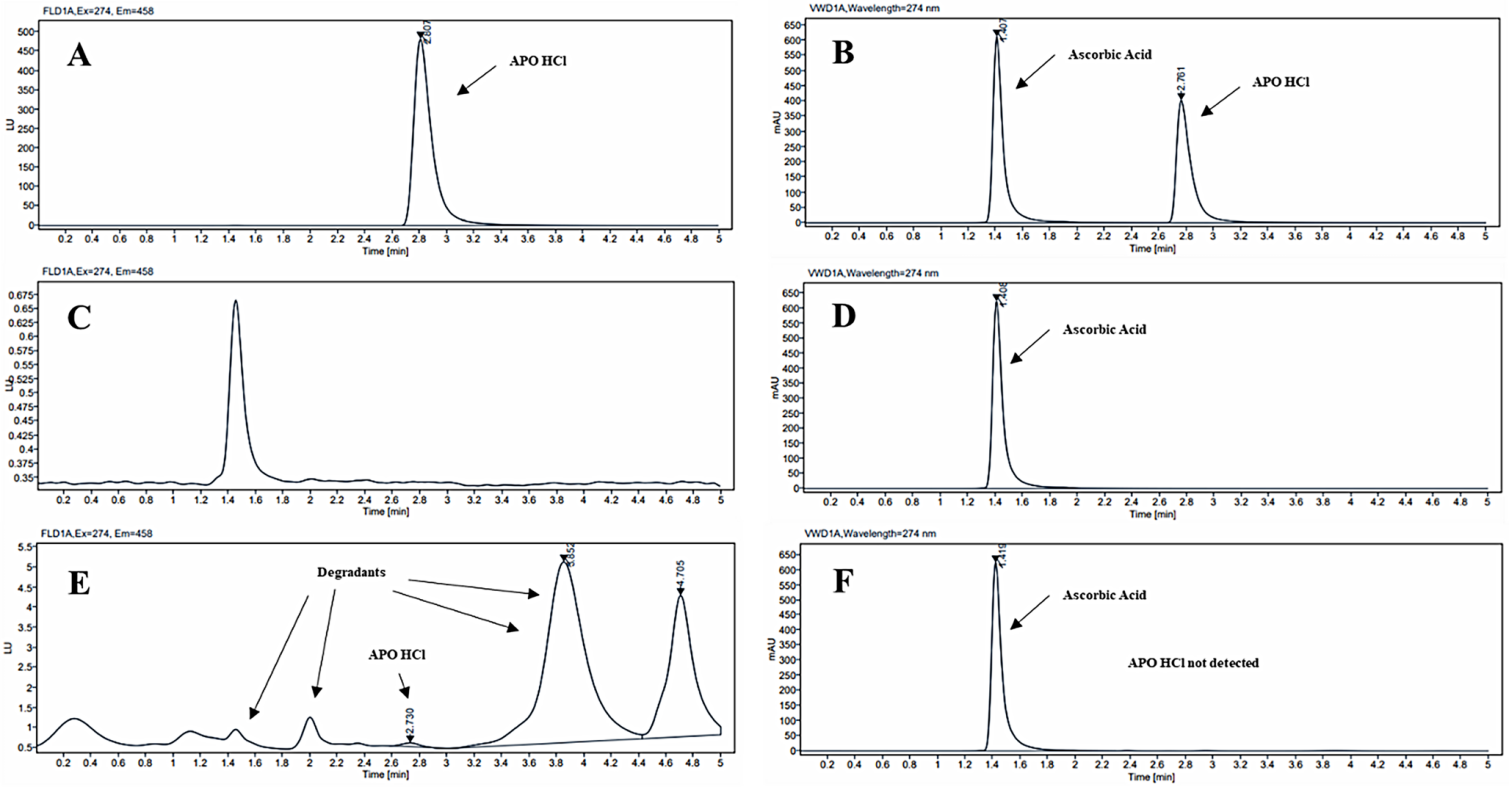
Chromatograms obtained *via* FID and UV arranged into left and right column, respectively. (**A**-**B**) 25 µg/mL APO prepared in porcine skin extract. (**C**-**D**) Blank porcine skin extract, with minimal to no interference in the retention time region of APO. (**E**-**F**) 100 µg/mL APO prepared in PBS (pH 7.4) and stored in 37 °C for 7 days, demonstrating baseline resolution from generated degradants labelled on chromatograms

Specificity of the RP-HPLC method to detect APO in presence of interferences derived from excised neonatal porcine skin and degradation is summarised in Fig. 5. In the retention time region of APO displayed in Fig. 5A and B (FID and UV detection respectively), there was minimal to no interference from porcine skin, as shown by Fig. 5D and E. Additionally, after 7 days storage at 37 °C in PBS (pH 7.4), without the presence of any stabiliser, APO nearly fully degraded, being in fact undetected by UV. However, the generated degradants did not elute in the retention time region of APO and were baseline resolved by both detection techniques, as confirmed by Fig. 5C and F, respectively. Thus, the method was stability-indicating, and can reliably quantify APO during ex vivo percutaneous permeation and deposition studies.

#### Selectivity

The RP-HPLC method demonstrated high selectivity, which in accordance with ICH M10 bioanalytical guidelines provided an LLOQ peak area response more than 5-fold greater than the response generated by blank porcine skin matrix, within the same retention time region. This is confirmed in Table 4, with the LLOQ: matrix ratio of both detection techniques being > 5. Comparing the two detection techniques, FID was unsurprisingly more selective than UV with average LLOQ: matrix ratios of 25.000 ± 4.696 and 7.084 ± 0.501, respectively, due to the 11-fold reduced response generated by the blank porcine skin matrix.

**Table 4:** Comparison of peak area responses of LLOQ QC standards versus blank Porcine extract (Means ± SD, *n* = 6)

Detection	Mean LLOQ response	Mean matrix response	Mean LLOQ: matrix
FID	7.614 ± 0.181	0.311 ± 0.043	25.000 ± 4.696
UV	24.981 ± 0.431	3.541 ± 0.253	7.084 ± 0.501

#### Within-run and between-run accuracy and precision

To assess the accuracy and precision performance of the RP-HPLC method, QC standards from four concentration levels were prepared based on the linear range of both detection techniques. Table 5 shows that repeated measurements of the high, medium and low concentrations of APO were consistently within ± 6% of the target value and maintained excellent precision within ± 10% CV, for both within-run and between-runs. Additionally, the LLOQ was shown to be well within acceptance limits of ± 20% bias and CV. Therefore, the RP-HPLC method can consistently provide accurate and precise data reliably over a period of time.

**Table 5:** Summary of within-run (*n =* 5) and between-run (*n =* 15) accuracy and precision studies (Means ± SD)

		Within-run			Between-run		
	Concentration of APO (ug/mL)	Peak Area (LU.s / mAU.s)	Bias (%)	CV (%)	Peak Area (LU.s / mAU.s)	Bias (%)	CV(%)
FID	10	836.453 ± 65.977	-2.48	7.87	860.268 ± 49.419	0.29	5.73
	5	410.962 ± 6.619	-3.97	1.60	423.114 ± 16.734	-1.14	3.94
	0.25	18.516 ± 1.994	-5.91	9.86	19.568 ± 1.454	-1.02	6.83
	0.098	7.300 ± 0.191	7.22	2.12	7.604 ± 1.044	10.85	11.22
UV	10	602.027 ± 48.601	-2.98	8.08	614.924 ± 33.687	-0.90	5.48
	5	296.466 ± 4.852	-4.55	1.64	304.893 ± 11.287	-1.83	3.71
	1	63.082 ± 1.570	0.77	2.51	63.389 ± 1.886	1.27	3.00
	0.391	27.556 ± 0.844	11.26	3.13	28.023 ± 0.840	13.19	3.06

#### Carryover

By comparing the blank response in Tables 4 and 6, there was notably an increased mean matrix response immediately after injection of a high concentration of APO (50 µg/mL). This was likely caused by a small amount residual analyte within or on the surface of the autosampler needle, resulting in minor carryover between injections. However, as confirmed in Table 6, the carryover was minimal and did not significantly affect the performance of the RP-HPLC method. Indeed, mean LLOQ: matrix response ratios of 7.79 ± 0.65 and 5.54 ± 0.03, for FID and UV detection respectively, were in accordance with acceptance criteria laid out in ICH M10 bioanalytical guidelines.

**Table 6:** Comparison between LLOQ QC standard response with response of blank Porcine skin extract directly after injection of 50 µg/mL APO (Means ± SD, *n* = 3)

Detection	Mean LLOQ response (LU.s / mAU.s)	Mean matrix response (LU.s / mAU.s)	Mean LLOQ: matrix
FID	7.647 ± 0.276	0.986 ± 0.085	7.79 ± 0.65
UV	24.992 ± 0.627	4.512 ± 0.022	5.54 ± 0.03

#### Dilution integrity

The dilution integrity was assessed using arbitrary dilution factors of 10 and 100, in order to give a general understanding of the effect the dilution process may have on the accuracy and precision of any quantitative measurements of APO. Table 7 shows that for both detection techniques, the % bias and CV of the diluted samples were will within acceptance criteria of ± 15%. Therefore, samples from ex vivo permeation or deposition studies may be reliably diluted to be within the linear range of the RP-HPLC method without affecting the integrity of the data.

**Table 7:** Analysis of the effects upon accuracy and precision of data caused by dilution of samples. Dilution factors of 10 and 100 were performed upon a 100 µg/mL APO sample (Means ± SD, *n* = 3)

Dilution Factor	Detection	Peak Area (LU.s / mAU.s)	Bias (%)	CV (%)
10	FID	904.54 ± 1.13	5.45 ± 0.13	0.12
	UV	650.97 ± 0.56	4.91 ± 0.09	0.09
100	FID	87.35 ± 0.89	3.61 ± 1.04	1.00
	UV	63.22 ± 1.54	0.99 ± 2.48	2.45

#### Robustness

In order to understand the robustness of the method to minor random variations in chromatographic conditions, these were purposefully performed with the intention of monitoring the effect upon quantitative measurement and elution time of APO. Table 8 highlights that slightly altering the mobile phase flow-rate, composition and TFA concentration will not produce any significant effect upon either of the aforementioned data parameters. Indeed, for both FID and UV detection, recovery of APO consistently remained between 96 and 102%, and retention time remained within ± 0.05 min of the original. Thus, the RP-HPLC method was shown to be highly robust, additionally due to the fact that it does not require any temperature control of the column compartment. Interestingly, this may highlight the ease-of-transfer of the method to an alternative HPLC instrument model or laboratory, and then employed by other analysts while maintaining performance. This is easily facilitated by the duality of both FID and UV detection.

**Table 8:** Summary of robustness studies; study of the effect caused by small alterations in % (v/v) TFA concentration in aqueous phase, % (v/v) ACN in mobile phase composition and flow-rate, upon APO peak retention time and response. (Means ± SD, *n* = 5)

		FID		UV	
Parameter	Setting	Recovery (%)	RT (min)	Recovery (%)	RT (min)
Flow rate (mL/min)	0.99	101.17 ± 0.08	2.70 ± 0.00	100.90 ± 0.06	2.66 ± 0.00
	1	100.00 ± 0.08	2.67 ±0.00	100.00 ± 0.04	2.62 ± 0.00
	1.01	99.11 ± 0.06	2.66 ±0.00	98.69 ± 0.04	2.61 ± 0.00
Mobile phase composition (%v/v)	70.1: 29.9	99.96 ± 0.04	2.70 ±0.00	99.47 ± 0.03	2.65 ± 0.00
	70.0: 30.0	100.00 ± 0.08	2.67 ±0.00	100.00 ± 0.04	2.62 ± 0.00
	69.9: 30.1	100.38 ± 0.08	2.67 ±0.00	99.24 ± 0.03	2.63 ± 0.00
% TFA (%v/v)	0.09	100.40 ± 0.07	2.66 ±0.00	98.01 ± 0.02	2.61 ± 0.00
	0.1	100.00 ± 0.08	2.67 ±0.00	100.00 ± 0.04	2.62 ± 0.00
	0.11	96.64 ± 0.04	2.69 ±0.00	96.94 ± 0.05	2.65 ± 0.00

### Stability studies and saturation solubility in PBS (pH 7.4) with 0.1% (w/w) AA

APO is known to rapidly auto-oxidise in aqueous solution to oxoapomorphine, particularly below pH 7 [27]. Of the previously published reports that study ex vivo permeation of APO across human or animal skin, some fail to account for this by omitting the use of antioxidants in their release media [15]. Additionally, those that do, employed sodium metabisulfite (SMB) to prevent chemical degradation of APO [18, 21, 24]. A recent study by Ang et al. provided an in-depth study on the stabilizing effect of different antioxidants including SMB and AA incorporated into acetate buffer (pH 4.0). Despite being commonly incorporated into formulated APO commercial products, SMB had a very weak capability of preventing auto-oxidation, and was outperformed by AA [27]. These findings indicate a flawed experimental design in the aforementioned studies. Thus, in order to select a more appropriate release media, AA was investigated further for its ability to stabilise APO when incorporated into PBS (pH 7.4).

**Fig. 6:**
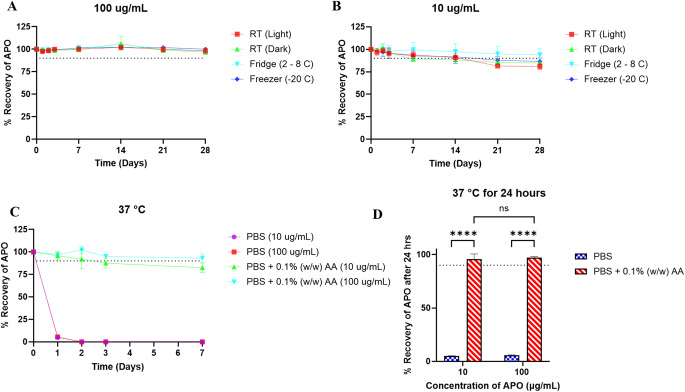
**(A**) and (**B**) show recovery of APO prepared in 0.1% (w/w) AA in PBS (pH 7.4) over a period of 28 days at two different concentrations (100 µg/mL and 10 µg/mL, respectively) when stored in various conditions. Dotted line represents 90% recovery (Means ± SD, *n* = 3). (**C**) compares recovery of APO stored in 37 °C over a period of 7 days when prepared in PBS (pH 7.4) with or without 0.1% (w/w) AA. Dotted line represents 90% recovery (Means ± SD, *n* = 3). (**D**) Direct comparison of recovery of APO after 24 h in 37 °C. Dotted line represents 90% recovery. (Means + SD, *n* = 3). All data shown in (**A** – **D**) quantified by FID

Figure 6A and B illustrate the recovery of APO over a period of 28 days in varying storage conditions, at two concentrations (100 µg/mL and 10 µg/mL). Clearly shown here is an inverse relationship between drug concentration and rate of degradation, driven by oxygen to drug ratio [27]. At high concentrations above 100 µg/mL, APO was stable in PBS (pH 7.4) with 0.1% (w/w) AA for up to 28 days even at room temperature in lit conditions. Although, reduced concentrations of APO may require lower temperatures during storage to maintain stability for long periods of time. Interestingly, APO had superior stability in refrigerated conditions, in comparison to -20 °C. Initially this seems counter-intuitive as storing pharmaceutical agents at temperatures < 0 °C is generally considered to enhance stability and obviate concerns regarding liquid storage. However, based on prior reports this phenomenon is not unprecedented and may be due to exclusion of drug solute molecules by the moving ice-front during the uncontrolled freezing process. This results in cryoconcentration of drug molecules into highly concentrated zones, which may have altered levels of freely available ascorbic acid to protect the APO from oxidative stress [34, 35]. Therefore, storing APO solutions dissolved in PBS (pH 7.4) with 0.1% (w/w) AA in refrigerated conditions of approximately 4 °C is recommended and may maintain > 90% recovery for up to 28 days.

The stabilising effect of incorporating 0.1% (w/w) AA into PBS (pH 7.4) is illustrated in Fig. 6C. Within 24 h at 37 °C, APO stored in PBS (pH 7.4) alone drastically degraded to as low as 5.60 ± 0.56% and 4.92 ± 0.47% of the initial 100 µg/mL and 10 µg/mL concentrations, respectively, and fully degraded after 48 h. However, when stored in PBS (pH 7.4) with 0.1% (w/w) AA, APO was stable for up to 48 h at 37 °C with > 90% recovery. Indeed, Fig. 6C illustrates that concentrations of 100 µg/mL or higher may even be stable for greater than 7 days at this temperature. Further highlighted in Fig. 6D, was significantly greater stability of APO within a 24-hour period, upon incorporation of 0.1% (w/w) AA into the PBS (pH 7.4) (*p* < 0.0001), which is more than sufficient for ex vivo percutaneous permeation and deposition studies, based on the limiting factor of porcine skin viability in non-refrigerated saline solutions [36]. Furthermore, no significant difference in recovery of the initial APO concentration was found between 100 µg/mL or 10 µg/mL after 24 h, suggesting that the oxygen: drug ratio effect was minimal within this period. Overall, PBS (pH 7.4) with 0.1% (w/w) AA may offer sufficient stability of APO throughout the duration and temperature conditions of typical ex vivo percutaneous permeation and deposition studies performed with neonatal porcine skin, thus ensuring reliable and accurate data. Additionally, these findings verify that choice of buffer may have a strong effect on the stabilising capability of antioxidants, as 0.1% (w/w) AA was shown to perform more effectively here when incorporated into PBS (pH 7.4) than with acetate buffer (pH 4.0), which was previously investigated and shown to have a negative impact on stability [27].

In order to maintain sink conditions, the concentration of drug in the release media contained in the receptor chamber of Franz diffusion cell apparatus should never exceed 10–30% of the saturation solubility [37, 38],. Through analysis by the validated RP-HPLC method, saturation solubility of APO in PBS (pH 7.4) with 0.1% (w/w) AA was determined to be 4.08 ± 0.20 mg/mL (*n* = 4). In addition to providing excellent stability, this media should easily achieve sink conditions for ex vivo permeation studies using modified-Franz diffusion cells, dependent on the drug-loading of the TDDS formulation applied in the donor compartment.

### Extraction efficiency of APO from porcine skin

**Fig. 7:**
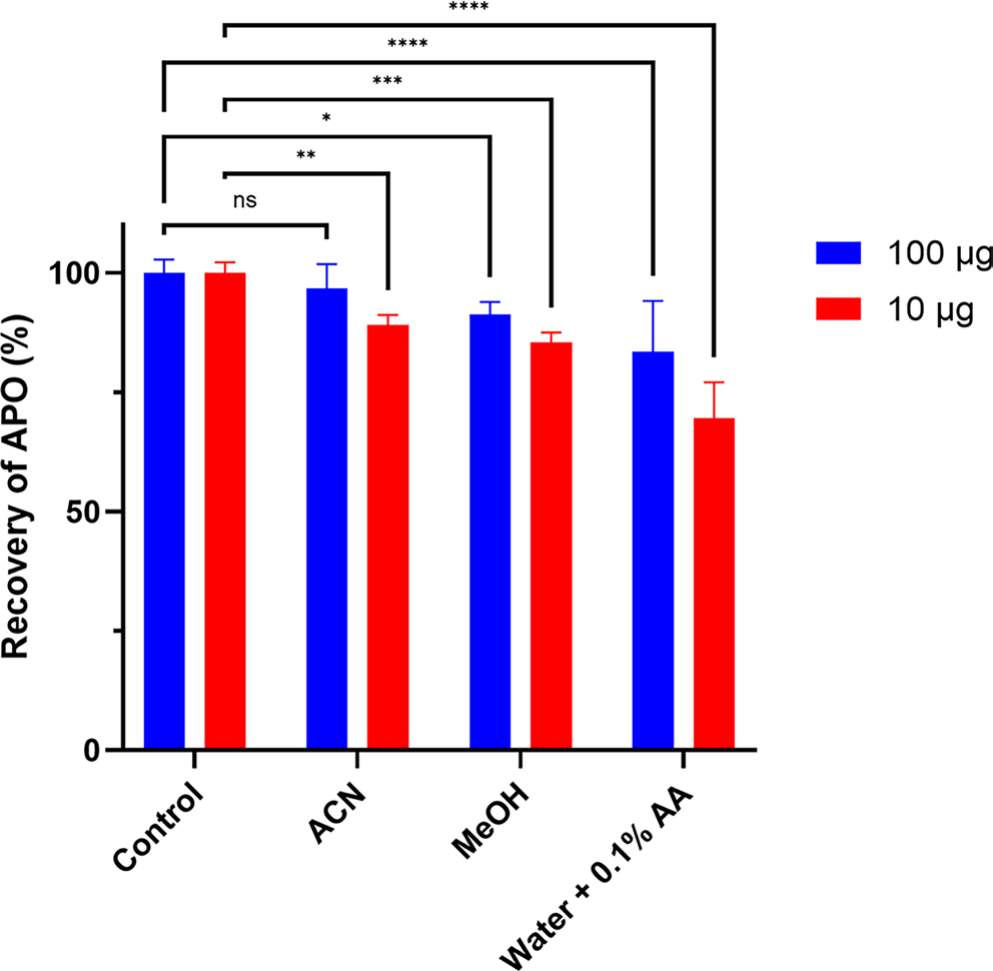
Efficiency of ACN, MeOH and 0.1% (w/w) AA in water to extract differing low amounts of APO deposited in full-thickness neonatal porcine skin. Quantified by FID (Means + SD, *n* = 5)

Summarised in Fig. 7 is a comparison of the efficiency of ACN, MeOH and 0.1% (w/w) AA in H_2_O, to extract low quantities of APO deposited within full-thickness neonatal porcine skin. These solvents extracted 96.66 ± 5.18% (no significant difference compared with control group) 91.24 ± 2.63% and 83.45 ± 10.67% of a 100 µg deposit, respectively. For a 10 µg deposit, each solvent had diminished extraction efficiency, with ACN, MeOH and 0.1% (w/w) AA in H_2_O demonstrating mean recoveries of 89.10 ± 2.05%, 85.34 ± 2.14%, and 69.59 ± 7.50%, respectively. Therefore, out of these solvents, ACN was proven to be the superior choice with the highest recovery of APO at both levels. Although, this process was concentration-dependent with lower concentrations of drug being harder to fully extract from porcine skin tissue, which may be partially due to the oxygen: drug ratio effect outlined previously, resulting in degradation during the extraction process. This may make factoring extraction efficiency into deposition studies challenging. However, ACN was able to essentially fully recover quantities close to and greater than 100 µg per 250 mg full-thickness neonatal porcine skin tissue (approximately 1 cm^2^), thus this may not be an issue in most cases.

### Determination of drug distribution of APO PSA films via multi-photon microscopy

**Fig. 8:**
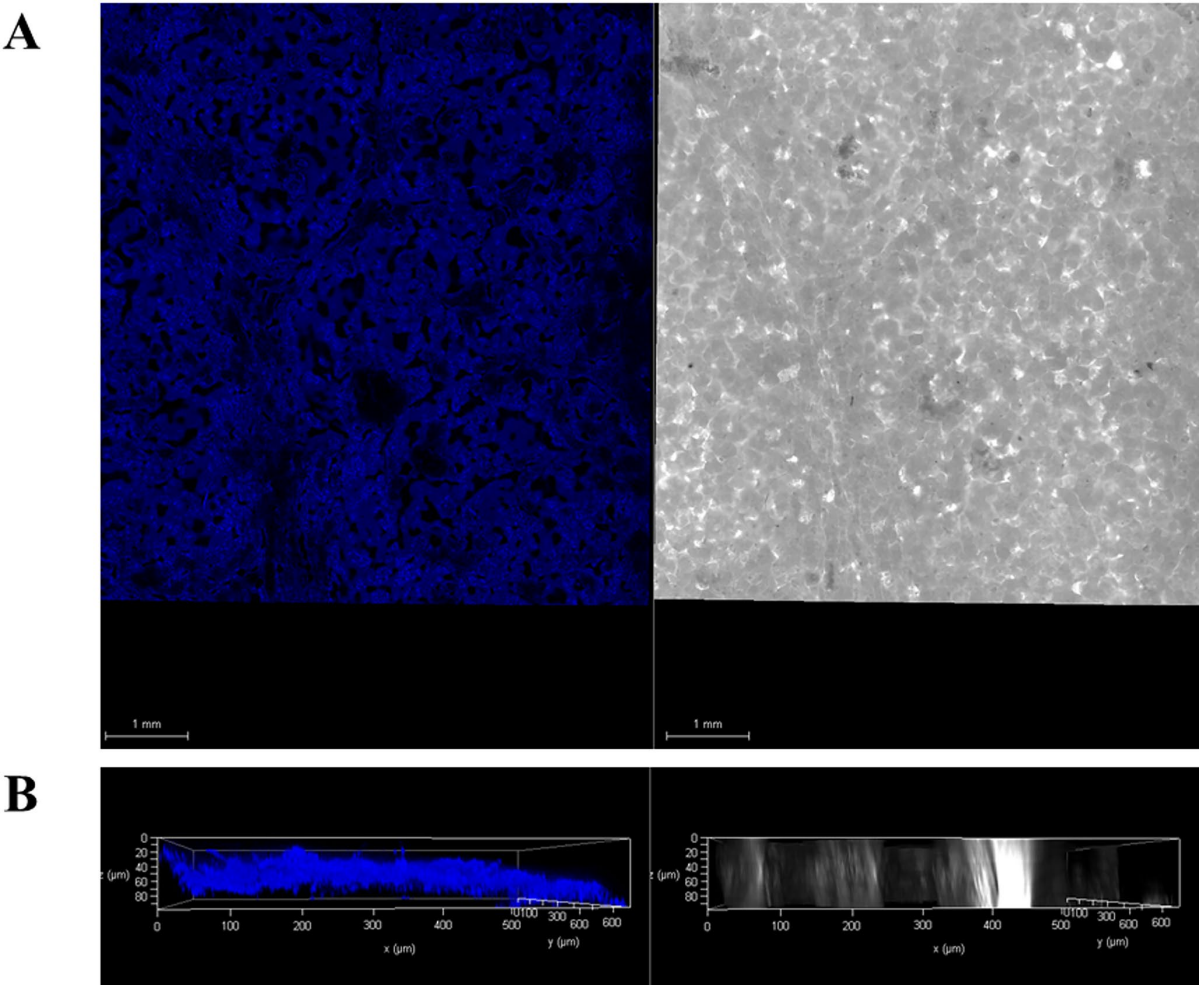
(**A**) Surface images of representative APO PSA film captured by TCS SP8 MP multi-photon microimaging. Left image shows distribution of APO (represented in blue) throughout patch surface. Right image displays visualisation of patch adhesive *via* light transmission. (**B**) XYZ stack of 600 × 600 μm area of patch surface, with APO highlighted in blue on left image

Due to the fluorescent nature of APO, the distribution of drug throughout the PSA films could easily be characterised using multi-photon microscopy *via* three-photon excitation at 822 nm, as shown in Fig. 8. The prepared APO PSA films were found possess uniform drug distribution throughout the patch adhesive, as illustrated in Fig. 8A, where the drug is highlighted in blue. Small voids were present with little-to-no APO; however, these were to be expected due to the drying process after solvent-casting [39]. The XYZ stack in Fig. 8B determined that APO was predominantly located 20–80 μm deep within the adhesive matrix of the PSA films. Overall, the PSA films were well-prepared with drug confirmed to be present throughout the entirety of the patch surface.

### Ex vivo percutaneous permeation and deposition study

**Fig. 9:**
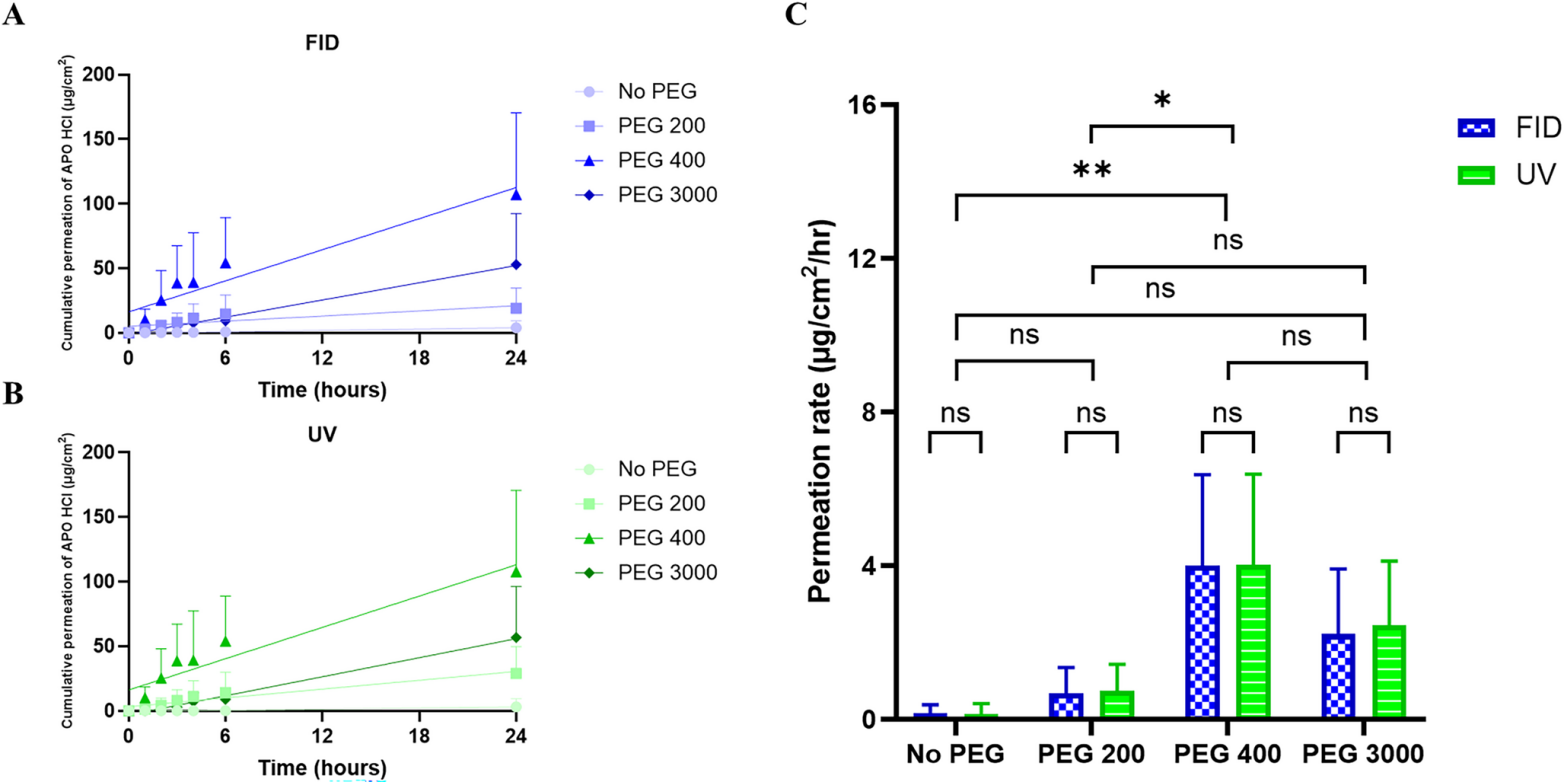
(**A**) and (**B**) show permeation profiles of each APO -PSA film formulation across dermatomed neonatal porcine skin over a 24-hour period, as detected by FID and UV respectively. Lines shown generated by simple linear regression. (Means + SD, *n* = 4). (**C**) Comparison of APO -PSA permeation rates determined from the slope of simple linear regression analysis (Means + SD, *n* = 4)

**Fig. 10:**
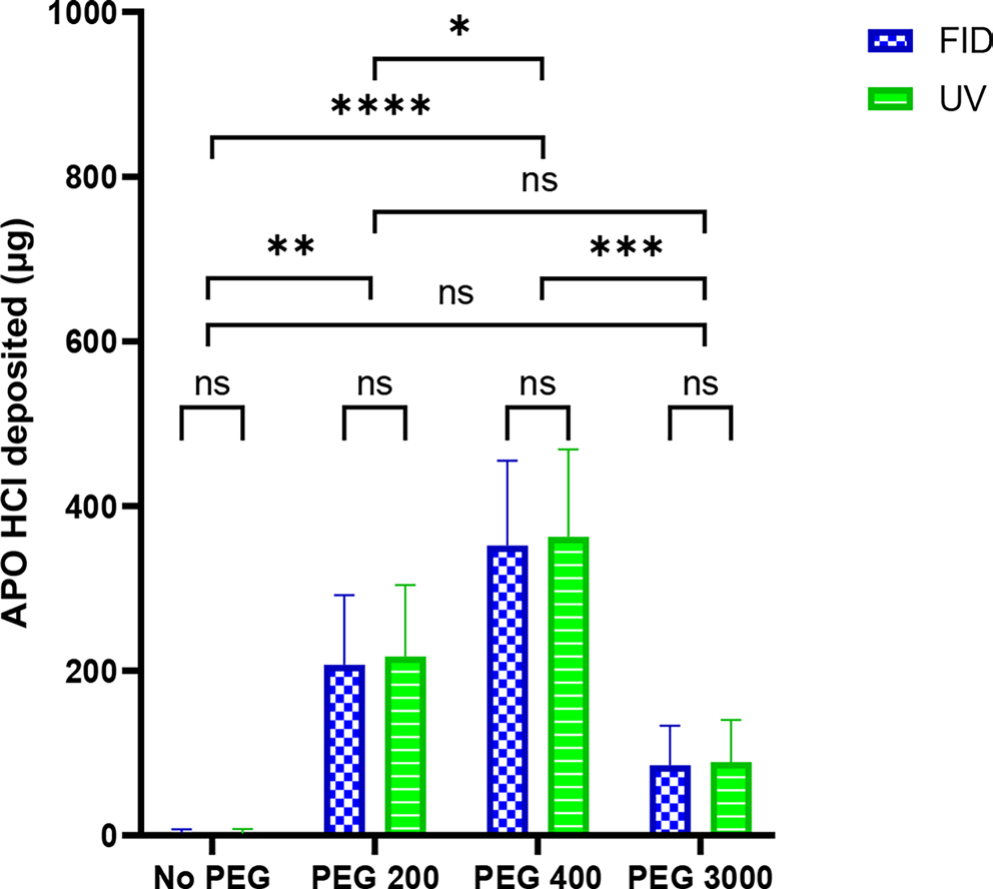
Comparison of deposition of APO in dermatomed neonatal porcine skin after 24 h adherence of APO-PSA films during ex vivo permeation study. (Means + SD, *n* = 4)

Demonstrated in Fig. 9 (A) and (B), is the novel approach of incorporating APO into 1 cm^2^ PSA films, which when used in conjunction with PEG penetration enhancers could successfully deliver APO across porcine skin. The effectiveness of the penetration-enhancing effect of PEG appeared to be molecular weight-dependent, with PEG 200, 400, and 3000 delivering 19.078 ± 15.590 µg/cm^2^, 107.020 ± 63.322 µg/cm^2^, and 52.872 ± 39.566 µg/cm^2^ over 24 h, respectively. Understandably so, as the lipid-fluidisation by nonionic surfactants is known to be optimal for carbon-chain lengths ranging between 10 and 14, similar to PEG 400 [40]. Each formulation generally followed Fickian-diffusion, demonstrating pseudo-steady state kinetics, allowing for the permeation profiles to be modelled by simple linear regression, with R^2^ values consistently > 0.89 as shown in Table 9 [41]. Thus, as shown in Fig. 9 (C), the flux of APO -PSA film formulations containing PEG 200, 400 and 3000 was 0.674 ± 0.678 µg/cm^2^/hr, 4.015 ± 2.361 µg/cm^2^/hr and 2.225 ± 1.699 µg/cm^2^/hr, respectively. Without the presence of PEG, APO could not effectively diffuse across dermatomed neonatal porcine skin, due to the physicochemical limitations of the drug molecule and the densely packed, lipid-rich extracellular matrix of the *stratum corneum* [42, 43]. Indeed, this was in line with prior reports showing poor transdermal permeation of APO without chemical modification, iontophoresis, microemulsion or microneedle mediated-techniques [15–24]. Therefore, the highest mean flux of APO across porcine skin was provided by films containing PEG 400, which was statistically significant compared to films manufactured with PEG 200 (*p* < 0.05) or without PEG (*p* < 0.01). However, no statistically significant difference was found between PEG 400 and PEG 3000 in terms of drug flux. It should be noted that, although necessary to ensure that uneven hair density and residual shafts do not compromise the effective contact area of the APO-PSA films, the pre-treatment of skin with depilatory hair removal cream may have minorly enhanced the permeation of APO. However, this effect will have been uniform across all of the formulations. Additionally, the occlusive conditions under which the study was performed will have resulted in increased hydration of the skin and thus an enhanced permeability of APO [44]. Deposition of APO in the dermatomed porcine skin was similarly affected by presence and molecular weight of PEG within the APO -PSA films, as highlighted in Fig. 10. Without the penetration-enhancing effect of PEG, APO almost completely remained within the adhesive matrix of the PSA films with only 3.156 ± 3.553 µg deposited in the porcine skin after 24 h application-time.

Based on their performance, these PSA films are unlikely to be clinically useful in delivering therapeutic doses of APO to patients suffering with PD. Indeed, they provided similar [15] or inferior [16–21, 23, 24] drug fluxes (per square unit area) in comparison to those aforementioned studies already in literature. This may largely be due to interactions between APO and carboxyl groups present in DuroTak^®^ 87-2677, resulting in inhibited skin permeation [45]. Therefore, to conclude that PSA films are not viable for APO may be premature, as the scope of this study provided merely an initial investigation into this novel approach to transdermally delivering APO, in order to demonstrate the applicability and versatility of the analytical methodology presented earlier. A more focused study into APO PSA patches should explore different adhesives, penetration enhancers and drug-loadings in order to provide a more complete assessment of the effectiveness of this approach.

Included in Figs. 9 and 10; Table 9 are comparisons in the results obtained *via* both UV and FID. Interestingly, both detection techniques provided highly similar data with no statistically significant differences found, thus highlighting the versatility of the analytical method. Furthermore, the high sensitivity of both detection techniques allowed for accurate quantification of low concentrations of APO in release media in the receptor compartment (< 6 h) and neonatal porcine skin. The RP-HPLC method described in the present work may therefore be reliably employed during ex vivo percutaneous permeation and deposition studies using either UV or FID, dependent on instrument availability, without compromising data integrity.

**Table 9:** R-squared values determined from linear regression analysis of permeation-time graphs shown in Fig. 6 (A) and (B)

	No PEG	PEG 200	PEG 400	PEG 3000
**Detection**	**R** ^**2**^			
FID	0.995	0.890	0.900	0.995
UV	0.944	0.910	0.903	0.992

## Conclusion

Presented here is a robust, versatile, validated analytical methodology for reliable and accurate quantification of APO deposited-within or permeated-through excised neonatal porcine skin, *via* either UV or FID, that may be easily transferred and adapted by future researchers for development of APO TDDSs. PSA patches composed of polyacrylate adhesive functionalised with carboxyl groups demonstrated applicability of the methodology, and provided minor percutaneous permeation of APO across neonatal porcine skin when used in conjunction with various molecular weights of PEG. Hypothetically, this may have been inhibited by interactions between APO and the adhesive, meaning further study into alternative PSAs, penetration enhancers and drug-loadings is necessary before concluding on the clinical potential of these systems.

## Data Availability

The datasets generated during and/or analysed during the current study are available from the corresponding author on reasonable request.
